# QualitySNPng: a user-friendly SNP detection and visualization tool

**DOI:** 10.1093/nar/gkt333

**Published:** 2013-04-30

**Authors:** Harm Nijveen, Martijn van Kaauwen, Danny G. Esselink, Brechtje Hoegen, Ben Vosman

**Affiliations:** ^1^Department of Plant Sciences, Laboratory of Bioinformatics, Wageningen University, PO Box 569, 6700AN Wageningen, The Netherlands, ^2^Netherlands Bioinformatics Centre (NBIC), PO Box 9101, 6500 HB Nijmegen, The Netherlands, ^3^Netherlands Consortium for Systems Biology (NCSB), PO Box 94215, 1090 GE Amsterdam, The Netherlands and ^4^Department of Plant Sciences, Plant Breeding, Wageningen UR, PO Box 386, 6700 AA Wageningen, The Netherlands

## Abstract

QualitySNPng is a new software tool for the detection and interactive visualization of single-nucleotide polymorphisms (SNPs). It uses a haplotype-based strategy to identify reliable SNPs; it is optimized for the analysis of current RNA-seq data; but it can also be used on genomic DNA sequences derived from next-generation sequencing experiments. QualitySNPng does not require a sequenced reference genome and delivers reliable SNPs for di- as well as polyploid species. The tool features a user-friendly interface, multiple filtering options to handle typical sequencing errors, support for SAM and ACE files and interactive visualization. QualitySNPng produces high-quality SNP information that can be used directly in genotyping by sequencing approaches for application in QTL and genome-wide association mapping as well as to populate SNP arrays. The software can be used as a stand-alone application with a graphical user interface or as part of a pipeline system like Galaxy. Versions for Windows, Mac OS X and Linux, as well as the source code, are available from http://www.bioinformatics.nl/QualitySNPng.

## INTRODUCTION

Recent developments in sequencing technology have revolutionized genetic research, as vast amounts of sequencing data are now becoming available. From this data, single-nucleotide polymorphism (SNP) information can be extracted that is useful for genetic analysis, including quantitative trait locus (QTL) mapping and genome-wide association studies (1,2). Although several tools for SNP detection are already available (3–5), they usually require Linux command line skills to run and use of a separate program to visualize the results. More user-friendly software would greatly benefit the community.

Since its publication, the QualitySNP pipeline for SNP detection in diploid and polyploidy species (6) has been successfully used in dozens of projects in plant and animal genetics, for instance, for the identification of SNP markers in crop plants (7), zebra finch (8), water fleas (9), snakes (10) and scallops (11). Because QualitySNP can use *de novo* assembled sequence alignments as input, it can also be used for species without a reference genome. The original QualitySNP was developed and optimized for Sanger sequenced expressed sequence tag (EST) data; however, the nature of DNA and RNA sequencing has changed drastically during the past 6 years, making an update necessary. Here, we present QualitySNPng that was specifically tuned to identify SNPs in data from the current next-generation sequencing (NGS) platforms. It features a graphical user interface (GUI), supports the popular SAM format (3), general performance improvements to allow analysis of large data sets and additional filtering parameters that address specific characteristics of NGS data from different platforms. The identified SNPs can be viewed in the context of predicted haplotypes and per input sample, making it ideally suited for genotyping by sequencing approach (1). Additionally, QualitySNPng can be used as a component in an analysis pipeline like the Galaxy platform (12).

## FEATURES

### SNP calling

QualitySNPng takes as input a sequence alignment file in SAM (3) or ACE (13) format with single-end or paired-end reads as produced by read mappers like Bowtie (14) and BWA (15) or *de novo* assemblers like CABOG (16) and PCAP (17). The QualitySNPng software uses three filtering steps to eliminate unreliable variations similar to the original QualitySNP (6). The first filter labels all nucleotide differences that occur in a minimum number of reads as potential SNPs. This minimum number can be adjusted by the user as an absolute number or a fraction of the total number of reads. The second filter takes into account the quality of the sequence containing the variant nucleotide and leaves only the high confidence SNPs. The base quality, characterized by the Phred score (18), is used for this when it is present in the input sequence alignment. If no Phred score is present, all nucleotides in the input reads are assumed to be of high quality. Additionally, the score can be modified based on specific sequence patterns. For instance, variations found in homopolymeric tracts can be set to low quality. This option is particularly useful when Roche/454 sequences are processed, as these are known to be prone to homopolymer-associated errors (19). Also a number of nucleotides at the 5′- or 3′-ends can be labelled as low quality, for instance to avoid false SNPs caused by incomplete adaptor trimming. The third filter involves predicting haplotypes based on the high confidence SNPs. Only if variation is supported by one or more haplotypes, it is considered as a reliable SNP. Compared with the original QualitySNP software, the second and third filters were reversed to make sure that the detected haplotypes are based on high confidence SNPs only. The run time largely depends on the size and nature of the input sequencing data, ranging from less than a minute for a set of ∼25 000 contigs (∼100 reads/contig) to 10 min for one large single contig of 7000 bp with 800 000 reads. Larger and more variable sequence alignments can take longer, also depending on the stringency of the settings: lowering the threshold for potential SNPs will result in more work for the second and third filters that are computationally the most expensive. For large input files that are expected to take several hours to process, one can use the command line ‘server mode’ option of the tool to perform the SNP calling on a compute server and subsequently analyse the results using the GUI.

### Viewing results

**Figure 1.**
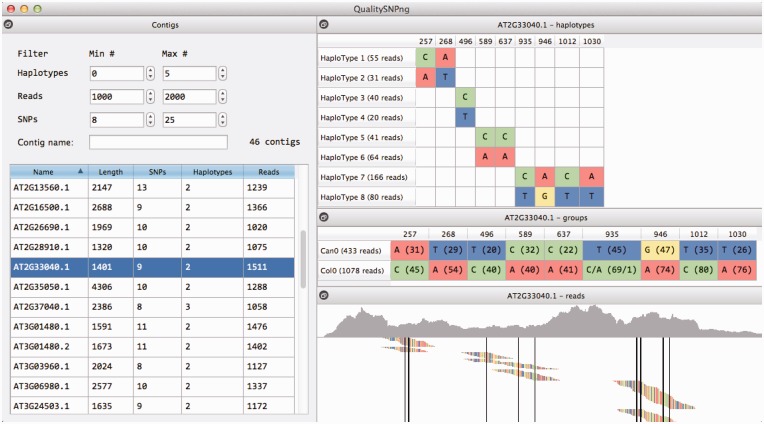
Screenshot of QualitySNPng output. Result of the SNP detection using *Arabidopsis thaliana* RNA-seq data set from two accessions that were mapped to *Arabidopsis* transcripts (20). In the left, the list of transcripts is shown, limited here using the filter options to only the ones with between 8 and 25 SNPs and between 1000 and 2000 reads. The details for the selected transcript are shown on the right: the top window shows the predicted haplotypes, the middle window shows the alleles per accession (Col-0 and Can-0) and the bottom window shows the reads aligned to the transcript sorted per haplotype (reads without SNP are not shown).

The results of the SNP calling can be viewed directly using the GUI, and they are also saved in structured text files for later reference or further processing. The different contigs from the input sequence alignments are listed in a table showing the number of SNPs, the reads and the haplotypes. The haplotype count in the table is corrected for fragmented haplotypes by taking the maximal number of haplotypes that is found per SNP position. Fragmentation of haplotypes may occur and is caused by SNPs that are too far apart to be linked to one allele by a single-sequence read or a read pair, see Figure 1 for an example. The contig list can be filtered based on the numbers of reads, SNPs and haplotypes and (partial) contig name.

A selected contig will show a window with the aligned reads and the SNPs indicated, a table with the haplotypes and their alleles per SNP position and a table showing the alleles for the different samples in the input data (Figure 1). For this last table to appear, the input sequence alignment file should be annotated with a ‘read group’ (see SAM format definition) per read, or alternatively, have group labels included in the read names. The overview per sample can for instance be used to compare alleles between different accessions, strains or ecotypes and for genotyping by sequencing.

Manual inspection of the read alignment together with the haplotype overview gives insight in the quality of the alignment, local read coverage and positions of the SNPs. Based on this visual inspection, one can decide to alter the stringency of the filter settings and rerun the SNP calling. The reads can be sorted on start position or per haplotype and can be viewed at different zoom levels.

For the creation of a SNP array, marker SNPs can be selected and exported with flanking sequence of a specified length as a structured text file that can be imported into a standard spreadsheet program or an assay design program.

To avoid problems in SNP scoring, we suggest selecting markers from contigs that have no more than the maximum expected number of haplotypes, i.e. two for diploid species, as contigs with more haplotypes may contain paralogous sequences. To further increase the chance of obtaining markers that will perform well on arrays, one could use the BLAST program (21) to eliminate marker sequences that show high similarity to other genes, as was shown previously (7).

## IMPLEMENTATION

QualitySNPng was written in C++ using the Qt toolkit. The same executable file can be used interactively with the GUI, or as a command line tool for inclusion in analysis pipelines to be run on a compute server. The software can be compiled and runs on the Windows, Mac OS X and Linux operating systems. The output data are saved as CSV text files and can be reloaded for later analysis using QualitySNPng, or processed by custom scripts for further analysis.

## DISCUSSION AND FUTURE DIRECTIONS

We believe there is a strong need for user-friendly software tools that allow biologists to directly analyse and visualize their data. QualitySNPng is a versatile tool that combines SNP detection and genotyping with interactive visualization of the results. The GUI with its pre-set filter options is easy to use and also highly configurable for specific needs. QualitySNPng is routinely used in-house for marker SNP identification in several projects (22–24). In one project, QualitySNPng was used to analyse RNA-seq data with up to 8 million reads per transcript to genotype a mixture of a few hundred accessions (unpublished) by making use of the ‘server mode’ option to run on a compute server. We expect that developments like in cloud computing will make this possible without leaving the GUI. The source code of QualitySNPng is freely available, and we encourage further development and implementation of the software in custom SNP analysis pipelines or adaptation for specific applications.

## FUNDING

The Netherlands Consortium for Systems Biology, which is part of the Netherlands Genomics Initiative/Netherlands Organization for Scientific Research. Funding for open access charge: Wageningen University and Research Centre.

*Conflict of interest statement.* None declared.
